# Identification of Stingless Bee Honey Adulteration Using Visible-Near Infrared Spectroscopy Combined with Aquaphotomics

**DOI:** 10.3390/molecules27072324

**Published:** 2022-04-03

**Authors:** Muna E. Raypah, Ahmad Fairuz Omar, Jelena Muncan, Musfirah Zulkurnain, Abdul Rahman Abdul Najib

**Affiliations:** 1 School of Physics, Universiti Sains Malaysia, Pulau Pinang 11800, Malaysia; muna_ezzi@usm.my (M.E.R.); abdulrahmannajib95@gmail.com (A.R.A.N.); 2 Aquaphotomics Research Department, Faculty of Agriculture, Kobe University, Kobe 658-8501, Japan; jmuncan@people.kobe-u.ac.jp; 3 Food Technology Division, School of Industrial Technology, Universiti Sains Malaysia, Pulau Pinang 11800, Malaysia; musfirah.z@usm.my

**Keywords:** adulteration, stingless bee honey, visible and near-infrared spectroscopy, PCA, PLSR, aquaphotomics

## Abstract

Honey is a natural product that is considered globally one of the most widely important foods. Various studies on authenticity detection of honey have been fulfilled using visible and near-infrared (Vis-NIR) spectroscopy techniques. However, there are limited studies on stingless bee honey (SBH) despite the increase of market demand for this food product. The objective of this work was to present the potential of Vis-NIR absorbance spectroscopy for profiling, classifying, and quantifying the adulterated SBH. The SBH sample was mixed with various percentages (10–90%) of adulterants, including distilled water, apple cider vinegar, and high fructose syrup. The results showed that the region at 400–1100 nm that is related to the color and water properties of the samples was effective to discriminate and quantify the adulterated SBH. By applying the principal component analysis (PCA) on adulterants and honey samples, the PCA score plot revealed the classification of the adulterants and adulterated SBHs. A partial least squares regression (PLSR) model was developed to quantify the contamination level in the SBH samples. The general PLSR model with the highest coefficient of determination and lowest root means square error of cross-validation ($R_{CV}^{2}=0.96$ and $RMSE_{CV}=5.88\;\%$) was acquired. The aquaphotomics analysis of adulteration in SBH with the three adulterants utilizing the short-wavelength NIR region (800–1100 nm) was presented. The structural changes of SBH due to adulteration were described in terms of the changes in the water molecular matrix, and the aquagrams were used to visualize the results. It was revealed that the integration of NIR spectroscopy with aquaphotomics could be used to detect the water molecular structures in the adulterated SBH.

## 1. Introduction

Honey is a natural food that may be used not only as a sweetener but also as a medication because of its therapeutic effects on human health. Flower honey is the most prevalent kind of honey, and it is made by honeybees (*Apis Mellifera* L.) from the nectar of various types of flowers, depending on the geographical region and season, while stingless bee honey is made from the nectar of trees and is a naturally higher moisture content [1]. Other compounds are often added to commercially sold honey for nutrition and medical purposes, and some are added to spoof the pure honey. Honey is often threatened by cheaper, commercially accessible sugar syrups with similar chemical compositions [2]. Even if these standards are followed to maintain high levels of quality, contaminations and/or adulterations might occur during manufacture, compromising the product’s quality and safety [3]. Adulterants in honey are any substances that are mixed with pure honey. Adulteration raises glucose levels, which can lead to diabetes, belly weight gain, and obesity. It also elevates blood lipid levels, which can lead to high blood pressure. Various adulteration activities of honey have been reported in the market, with the most common being the addition of edible syrups such as high-fructose corn syrup and corn syrup [4]. Many approaches, both traditional and modern, have been used and developed to distinguish pure honey from contaminated or non-honeys [5].

Spectroscopy has been more popular in the detection of honey adulteration in recent years due to its speed, simplicity, environmental friendliness, and nondestructive nature. It’s worth noting that strategies for proving the authenticity of products are always evolving in response to these increasingly widespread behaviors [3]. Recently, several spectroscopy methods, such as Raman spectroscopy [6], NIR spectroscopy on *Apis mellifera* [7,8,9], and stingless bee honey [1,9] were conducted to determine the adulteration. The combination of NIR spectroscopy application and chemometric methods [10,11] shows a simple, rapid, and nondestructive method on adulterated honey. Moreover, honey consistency is possibly fluid, viscous, or partially to completely crystalline [12]. Because hydrogen bonds, which are associated with water, are present in most natural samples, this method can be used in a variety of domains [9]. In the research of adulteration detection in honey, spectroscopic techniques such as near- and mid-infrared spectroscopy were used [4]. Researchers are constantly developing efficient and sensitive detection methods using NIR spectroscopic approaches to detect adulterants in honey [13]. At present, honey adulteration is generally detected on the bees’ honey. Stingless bee honey has a high-water content, slight sweetness, acidic flavor, fluid texture, and slow crystallization [14]. Compared to bees’ honey, stingless bee honey contains a higher amount of water, ranging between 25 to 56 g/100 g, and higher acidity, which influences its shelf-life and keeping quality. Honey produced by stingless bees (Melipona sp. and Trigona sp.) is recognized for its unique sour taste and odor [15]. Based on the local beekeepers’ report, the adulteration of SBH involves dilution with water to increase the yield volume of the honey to fraudulently increase income. In addition, in certain cases, cheaper honey is blended with a sour-tasting liquid such as vinegar to create a sour taste that resembles that of SBH, and then market it at a high price commensurate with the authentic SBH [16]. However, due to the complexity of food matrices, most standard approaches can only detect a small number of adulterants in stingless bee honey without a thorough examination of all of its attributes. Hence, less research is available on the adulterated stingless bee honey.

After carbohydrates, water is the second-largest component of honey and is considered as one of the most important features that affect several characteristics of honey, such as viscosity, flavor, specific weight, shelf-life, and crystallization [17,18]. In the case of stingless bee honeys, the water content ~ 20% is generally higher compared to the genus Apis and can be even higher than 40% depending on the bee species [19]. As a result, water is a very significant component that should be considered during the analysis of SBHs. It is well-known that different water molecular structures can be identified based on their respective absorbance bands in the NIR region, providing the NIR spectroscopy with the ability to detect subtle changes associated with water molecular structure changes. This is especially utilized in aquaphotomics [20,21], a spectroscopy-based science that employs the structure of water in bio-aqueous systems to interpret their functionality, and the results can be visualized using simple and quick tools called aquagrams [22,23]. Recent studies applied aquaphotomics for the detection of honey adulteration in which the structural changes of honey upon adulteration were explained in the terms of the changes in the water molecular matrix [9,13].

Apart from the common type of honey that is produced by bees of the genus Apis, demand for the honey produced from the stingless bee genus has increased recently and carries commercial value higher than the one of the genus Apis. However, despite holding increasing market demand, there are fewer studies and literature available regarding stingless bee honey’s (SBH) physicochemical properties, particularly its optical visible and near-infrared (Vis-NIR) properties. SBH has limited industrial production, lower shelf life, and lack of an institutional quality standard, due to the scant knowledge about the product and the distribution limitation of SBHs in the world market compared with honey from the genus Apis [24]. This deficiency of information weighs down the definition of quality attributes and standards concerning SBHs. Most of the physicochemical characteristics of honey available are related to beehive products directed to Apis products [25]. As a result, SBH is not controlled by food control authorities, since it is not included in the international standards for honey [26]. For that reason, consumers have no quality assurance on SBH products [25,27]. In other words, the lack of comprehensive physicochemical data allows the recognition of possible adulteration activities to become even harder to implement for SBH. Hence, this research is designed to quantify the alteration in the Vis-NIR spectra of the SBH concerning adulteration by different percentages of added water, apple cider vinegar, and high fructose syrup. In addition, multivariate analysis including principal component analysis (PCA) and partial least squares regression (PLSR) is performed on the Vis-NIR spectroscopy in the region of 400–1100 nm. What is more, the analysis of the adulterated SBH using aquaphotomics is presented. To the best of the authors’ knowledge, this is the first time to evaluate the adulterated SBH using the combination of aquaphotomics and NIR spectroscopy in the region 800–1100 nm.

## 2. Results and Discussion

### 2.1. Vis-NIR Spectra of Pure and Adulterated SBH Samples

**Figure 1:**
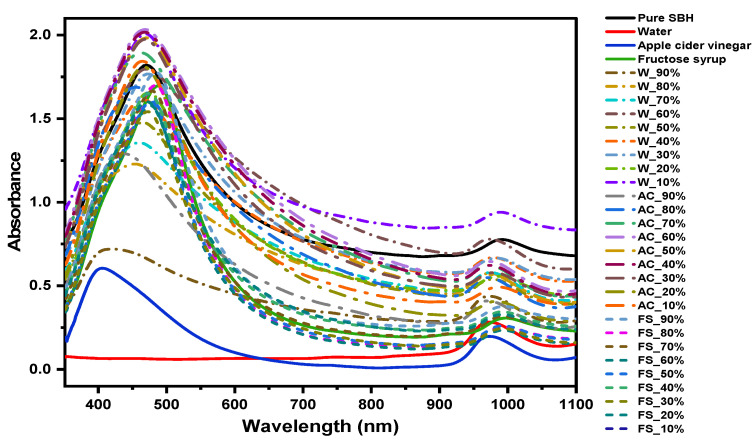
Spectra for pure SBH, adulterants, and adulterated SBH samples (*n =* 31).

The raw spectra of the samples (*n* = 31) are presented in Figure 1, and the adulterated samples are labeled by the type and percentage of adulteration. The spectral profiles in the Vis-NIR region showed three distinctive groups, where fructose-syrup (FS)-adulterated honey samples are particularly different compared to the water (W)- and apple-cider (AC)-adulterated honey. Depending on the adulterant type, there is a vastly different baseline offset, an effect that can be attributed to light scattering due to the sugar crystals present in the honey samples [28,29]. Alternatively, an increase in the baseline could be explained by increased bulk and hydration water content, that causes an increase in the scattering [23,30,31]. Most probably, the two phenomena are inter-connected, suggesting an altered water–sugar interaction by adding apple cider and water that results in changes in the crystallization degree.

**Figure 2:**
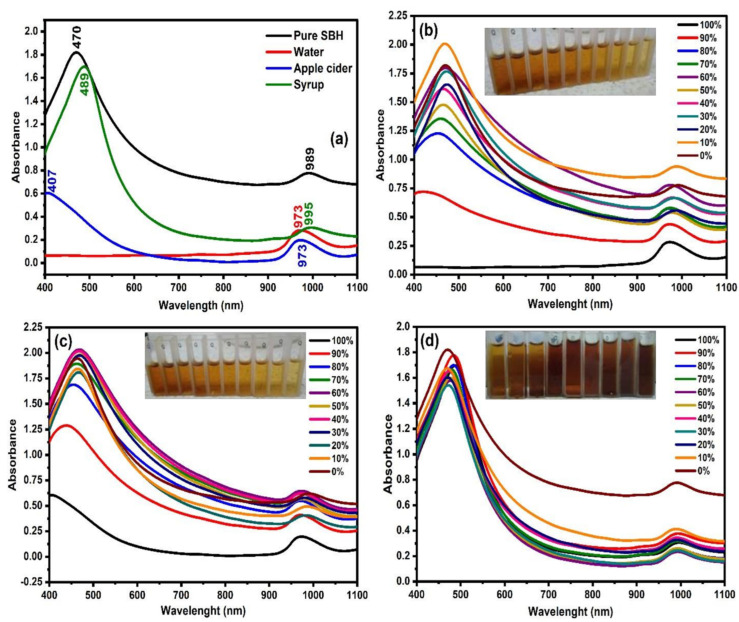
Vis-NIR spectra of (**a**) Pure SBH and adulterants and adulterated SBH by different percentages of water (**b**), apple cider (**c**), and fructose syrup (**d**).

The Vis-NIR spectra (400–1100 nm) of pure SBH and the adulterants together with SBH adulterated with various percentages of each adulterant are shown in Figure 2. It is apparent from Figure 2 that the baseline offset shows the largest differences depending on the percentage of added adulterant in the spectra of water-adulterated honey, followed by apple cider and the smallest variations can be observed in the fructose-syrup-adulterated honey samples. Interestingly, the spectrum of pure honey is furthest away from the fructose-syrup-adulterated honey spectra. While the changes in the color of the honey may be subtle, as can be seen in the visible part of the spectrum, the observed spectral characteristics in the NIR region of the spectra suggest a change in other optical properties that may be connected to the scattering of light, for example, the refractive index [32], or the optical rotation, as was shown to be the case in other honey adulteration studies [33,34,35].

As can be seen in Figure 2a, there are differences in intensity and wavelength peak between samples in the visible region. The peaks at a visible region for the pure SBH, apple cider, and fructose syrup are located at 470 nm, 407 nm, and 489 nm, respectively. The color of honey is the first quality aspect that affects consumer predilection. The color of honey is related to the floral origin or the plant source, minerals, phenolic contents, storage time, and temperature [36]. The absorbance at 400–500 nm is related to the honey compounds that absorb the light, such as the blue–violet light range at 400 nm, that resulted in the characteristic orange-amber color of honey [37]. On the other hand, some intensity differences can be observed in the visible region at 400–700 nm. The higher absorbance intensity designates the dark color of the sample [38]. The pure SBH spectrum presented the highest intensity in this range, followed by apple cider vinegar and fructose syrup. Pure honey remained in the middle range, with its orange-amber color, followed by the apple cider and fructose syrup, that exhibited different colors. The band around the region between 550 nm and 600 nm characterizes the maximum emission of riboflavin (vitamin B2) presented in the samples [39].

Likewise, the raw spectra in Figure 2a demonstrated that the absorption peaks of the samples are mainly located in the NIR region. The peak of absorbance within the NIR region for water and apple cider is located at 973 nm, which is associated with the second overtone of the symmetric and asymmetric OH-stretching bands [40,41,42]. The apple cider generally contains up to 94% of water content [43]. The NIR absorbance for fructose syrup is further shifted to a higher wavelength with a peak of 995 nm, while the NIR peak for pure SBH was recorded at 989 nm. The determination of the original Vis-NIR properties of the samples is important in setting the potential benchmarking on the authenticity of the SBH, thus allowing the detection—and, preferably, the quantification—of the adulterant through the alteration on the spectral attributes of the sample. Figure 2b–d shows the Vis-NIR spectra of SBH adulterated by different percentages of water, apple cider, and fructose syrup, respectively. The adulteration is shown at an increasing percentage from 10 to 90%, while 0% refers to the spectrum of pure SBH and 100% to the spectrum of the adulterant. The color of pure SBH can range from white to dark amber [44,45]. From the images of the samples, the SBH adulterated with water and apple cider exhibits visible color changes from light amber to water white (based on the Pfund scale) [46,47]. However, the SBH sample adulterated with fructose syrup does not show significant changes from the visual perception.

Spectroscopic and colorimetric techniques can provide a more exact evaluation of honey color and are used to identify the small differences between the color of honeys [47]. A further possible practice for the determination of honey color is using the colorimetric parameters based on spectral information. This can be achieved in a chromaticity diagram (or a color space) developed for the perception of the human eye, such as the CIELAB/CIE XYZ/CIELUV color space. In the case of honey analysis, the CIELAB color space is the most widely applied [2,36,48,49], used in this study concerning illuminant D65 and a visual angle of $10^{{}^{\circ}}$ [48,49]. The absorbance spectra of the samples were transformed to transmission spectra using the Unscrambler Software and transferred to a Chromaticity Diagram template developed in the OriginPro 2021 Software. It must be highlighted that colorimeters apply continuous filters and provide the X, Y, and Z tristimulus values as output, while spectrometers measured the transmission/reflection/emission spectra. Since color parameters values are derived from the spectral data, the results may carry the error caused by the spectral resolution of the spectrometer.

Within the approximately uniform CIELAB color space, two-color coordinates, $a^{*}$ and $b^{*}$, as well as lightness $L^{*}$ are defined. $L^{*}$ is an approximate measurement of the degree of lightness within the range of 0–100 (0 for black till 100 for white). The $a^{*}$ coordinate determines the greenness/redness of the sample. The greenish color is in the negative range, while the reddish color is in the positive range. The $b^{*}$ coordinate indicates the blue or yellow color, where blueness is in the negative range and yellowness in the positive range. It was found that water and apple cider were the lightest by visual comparison and have the highest values of the parameter $L^{*}$, that indicates the lightness—94.55 and 84.12, respectively. The $L^{*}$ value decreased further in pure SBH (30.08) and fructose syrup (46.35). The SBH in this study can be considered as dark honey, since $L^{*}\leq \;$50 [36]. This could be attributed to the floral source, the species of bee, and the location of honey production. It was shown that the honey with the highest levels of total minerals exhibit quite low values of $L^{*}$, coinciding with a dark color [49]. Furthermore, these results were in close relationship with the absorbance spectra shown in Figure 2a and confirm that the absorbance increased with the decreasing lightness value, as reported by Bertoncelj et al. [36]. What is more, the CIE XYZ (or CIE 1931) chromaticity diagram is displayed in Supplementary Figures S1 and S2. In these figures, the adulterated samples were labeled as H_W9 to H_W1, H_C9 to H_C1, and H_S9 to H_S1, which means the honey is adulterated with water, apple cider vinegar, and fructose syrup from 90% to 10%, respectively. The chromaticity diagram (Figure S1) and its zoom-in view (Figure S2) proved the variation in the value of $L^{*}$ due to the addition of adulterants, which is related to the interpretation in Figure 2.

**Figure 3:**
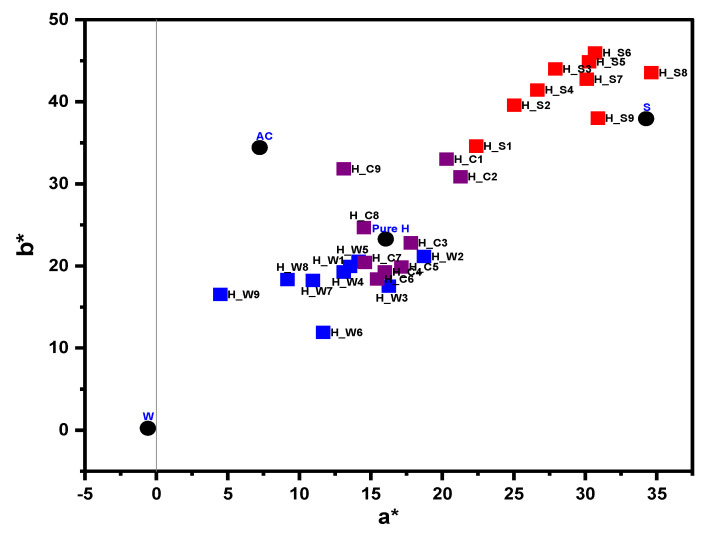
Location zone of adulterants and honey samples in the ($a^{*}$, $b^{*}$) diagram.

The plot of parameters $a^{*}$ and $b^{*}$ of the samples is shown in Figure 3. From the diagram, it is apparent that, depending on $L^{*}$, the samples were clearly distinguished from each other, as it was more obvious in adulterants and pure SBH. Additionally, the adulterated honey with fructose syrup is clearly distinguished from the other adulterated samples. Likewise, at a specific level of adulteration, the adulterated honey samples due to water and apple cider vinegar at a specific range of $a^{*}$ and $b^{*}$ are overlapped in the ($a^{*}$,$\;b^{*}$) scheme, resulting from their similar color characteristics.

**Figure 4:**
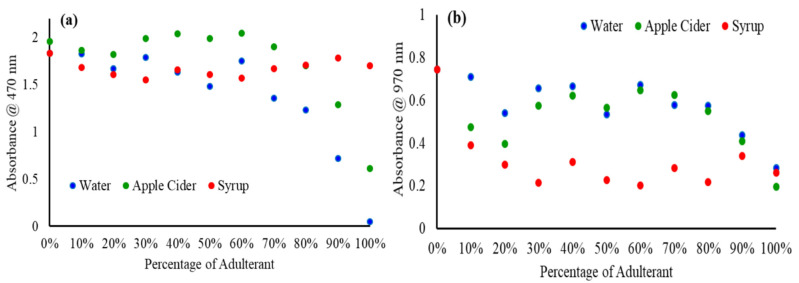
Relationship between the percentage of added adulterant and absorbance at (**a**) 470 nm and (**b**) 970 nm.

The Vis-NIR spectra can be characterized with two main peaks, at 470 nm and 980 nm, for the SBH as reported earlier [50]. The high fructose syrup showed a similar peak characterization, at 500 nm and 980 nm. On the other hand, apple cider also showed two main peaks at 400 nm and 970 nm, while water only exhibited a peak at 970 nm. In order to be able to quantify the spectral alteration due to the added adulterant in the pure SBH sample, the relationship between the absorbance at 470 nm and 970 nm with the percentage of adulterant was plotted and is shown in Figure 4a and b, respectively. In general, there is no substantial degradation in the value of absorbance for SBH adulterated with 10 to 60% of water and apple cider. The spectral degradation begins to take place when the quantity of adulterant is raised to 70% onwards. This observation was found for both 470 and 970 nm. As presented in Figure 4, the relationship between the concentration of adulterant and absorbance can be quantified by a linear correlation coefficient (R), where the R values of 0.8456 and 0.6653 were generated between the absorbance at 470 nm with the percentage of water and apple cider, respectively. In addition, the R of 0.7295 was generated between the absorbance at 970 nm with the percentage of water, but a very low R, of 0.3716, was produced for the apple cider. Nevertheless, at 470 nm, the SBH adulterated with fructose syrup showed no notable correlation with the spectral absorbance, with data fluctuating between 1.548 and 1.822 with a standard deviation of 0.073. For a similar dataset, the absorbance at 970 nm dropped 0.353 points from 0.743 when the pure SHB sample was added with 10% of syrup. However, the continuous addition of fructose syrup did not cause further reduction in the value of absorbance, where the data only fluctuated between 0.202 and 0.39 with a standard deviation of 0.058.

**Figure 5:**
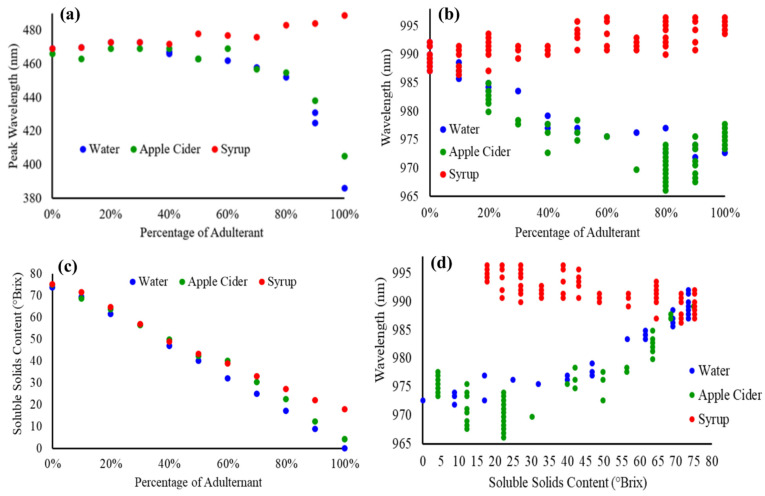
(**a**) Visible and (**b**) near-infrared peak absorbance of the samples against the percentage of added adulterant, (**c**) relationship between soluble solids content (in ^o^Brix) and the percentage of added adulterant, and (**d**) absorbance at near-infrared peak and the soluble solids content of the samples.

The peak shift in the Vis and NIR spectra towards the percentage of added adulterant was also investigated. From Figure 5a, for the visible region, a hypochromic shift occurs from 469 nm to 386 nm for water and from 466 nm to 405 nm for apple cider. SBH adulterated with fructose syrup, on the other hand, causes a consistent bathochromic shift from 469 nm to 489 nm, with the increment of added adulterant. The shift in the wavelength is predominantly from 50% of added adulterants. The pattern of the response curve is almost similar to Figure 4a due to the transformation in the SBH color caused by the adulteration. Water and apple cider cause the SBH samples to lose their yellow-red property, while the addition of fructose syrup enriched the yellow-red properties of the SBH due to the original color properties of the fructose syrup (as can be seen in Figure 2). 

The relationship between the wavelength of the NIR peak and the amount of the adulterants in SBH samples is depicted in Figure 5b. Generally, the NIR region exhibits a broad peak where multiple wavelengths share similar maximum values compared to visible absorbance. This is more significant for SBH samples adulterated with apple cider and fructose syrup. The NIR peak absorbance for pure SBH is located between 987 and 992 nm. The NIR peak shifted significantly to a lower NIR wavelength until it reached the centralized water peak at 970 nm with the increase in water and apple cider. A previous study reported that the combination of NIR wavelengths between 960 and 965 nm within C-H and O-H bands can reliably quantify sugars including fructose, glucose, and sucrose [51]. 

Generally, the addition of water and apple cider (predominantly composed of water) reduced the values of the soluble solids content ‘SSC’ comparably, while the addition of high fructose syrup increased the SSC further, especially at concentrations beyond 60%, as shown in Figure 5c. Pure SBH has an average SSC of 74.5 ^o^Brix. According to a review written by [44], the SSC for SBH ranges from 64.5 to 75.8 ^o^Brix and is lower than the one for Apis mellifera honey due to its higher water content and lower sugar content. The sugar composition of SBH was recently discovered to be predominantly trehalulose, which explains its lower total fructose and glucose contents [52]. The pure apple cider recorded 4.1 ^o^Brix contributed by sugar and organic acids. Pure high fructose syrup, on the other hand, recorded a much higher SSC, at 17.8 ^o^Brix.

The wavelength of the NIR peak was plotted against the soluble solids content of the samples, as shown in Figure 5d. The increase in ^o^Brix value showed a gradual increment of the NIR wavelength beyond 970 nm for both samples adulterated with water and apple cider. These results are in agreement with some studies in which the experiment was conducted to analyze the NIR properties of water-sugar solutions [51,53,54]. In these studies, raising sugar concentration in water (i.e., high SSC) causes the water band to be more symmetric and, as a result, shifts the peak absorbance towards the longer wavelength. Omar et al. [50] also reported that the addition of water to both SBH and Apis mellifera honey resulted in a shift of the NIR absorbance peak towards a lower wavelength. However, the increase in ^o^Brix value of the SBH that was adulterated with syrup exhibits a very minimal shift towards a lower wavelength for the higher SSC of the adulterated samples.

### 2.2. PCA Analysis and PLSR Modeling

**Figure 6:**
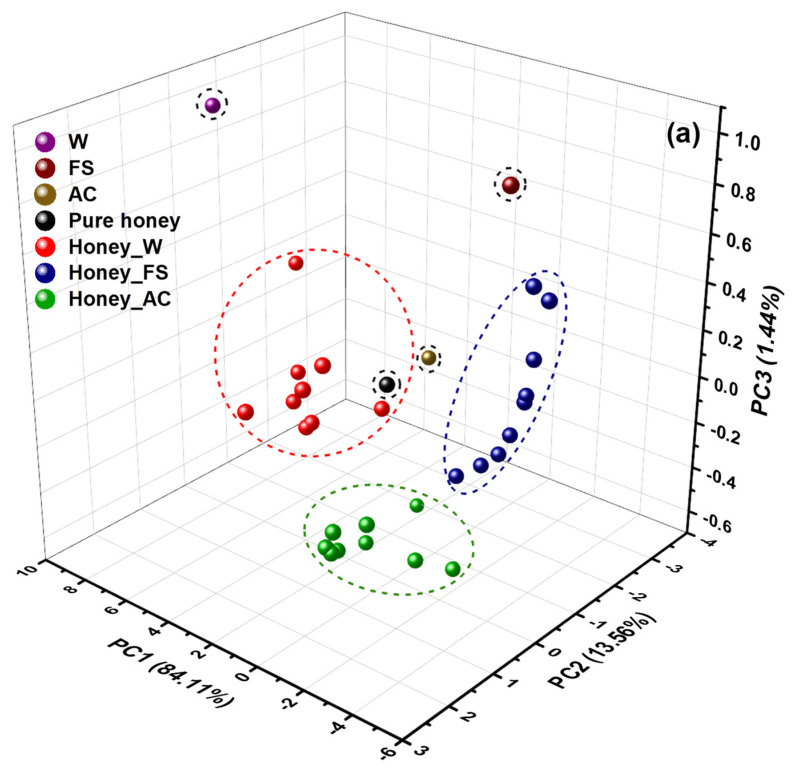

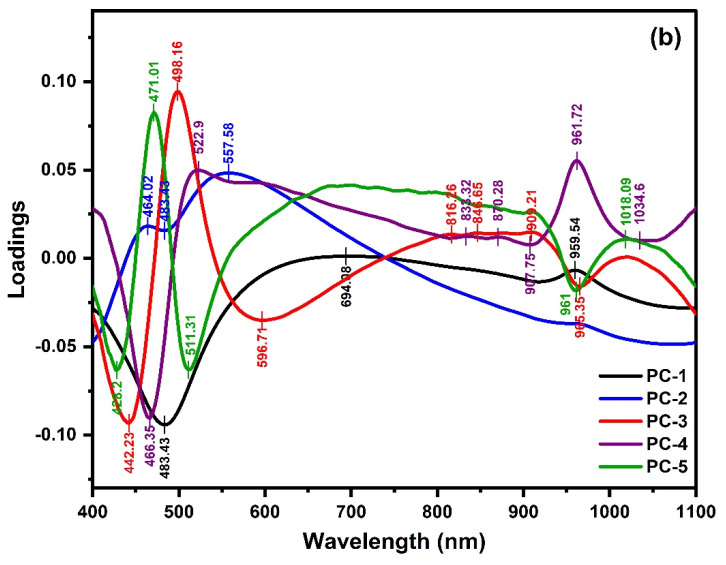
PCA analysis: (**a**) 3D score plot of the first three PCs and (**b**) x-loadings of PC1-PC5 of Vis-NIR spectra (400–1100 nm).

Room temperature Vis-NIR spectra of pure and adulterated honey were exported from the spectral software system and introduced directly into the Unscrambler software to generate the chemometric models. A PCA was performed on the Vis-NIR spectra (400–1100 nm) due to its ability to decrease dimensionality. To enhance the spectral quality and attain a good clustering for samples, the raw data were first pre-processed using moving average smoothing (within a 25-segment size) and baseline transformations. The 3D score plot for all the samples according to the first three principal components (PCs) is shown in Figure 6a. 2D PCA score plots of PC1 vs. PC2, PC1 vs. PC3, and PC2 vs. PC3 are presented in Supplementary Figure S3–S5, respectively. As presented in Figure 6a, the samples were clustered into seven groups, that corresponded to the three adulterants (water ‘W’, apple cider ‘AC’, and fructose syrup ‘FS’) and four groups of SBHs (pure and adulterated honey). The honey samples were clustered into different groups and zones based on the presence of adulterants, suggesting that these samples have distinctive properties concerning the adulteration type. In addition, the cumulative explained variance of the first three PCs was 99.11% of the total variance of spectral data, which indicates that these PCs can reflect most of the basic characteristics of the raw data.

The PCA loadings reveal the wavelengths that show the maximum difference at the individual PCs [55]. The loadings of each wavelength to differentiate the SBHs were evaluated and displayed in Figure 6b for the first five components (PC1 to PC5). Several contributive wavelengths with high x-loadings are recognized at Vis and NIR regions. These wavelengths including 442, 466, 471, 483, 498, 511, 558, and 597 nm in the Vis region, and 816, 833, 847, 870, 908, 909, 960, 961, 965, 1018, and 1035 nm in the NIR region. The peaks at the visible region are related to the color of the honey and the effect of the adulterations’ type and level on the color of honey samples, while the peaks in the NIR region are located within the areas of the third overtone of the C-H stretching band [56,57], the second overtone of the OH stretching band of water [9,58,59], and the second overtone of the N-H stretching band. These spectral features arise from the major components of honey, including carbohydrates, water, proteins, and amino acids [60]. The findings demonstrated that the potential adulteration of SBH by common adulterants such as water, apple cider, and high fructose syrup can be discriminated with a clear separation. Despite the complexity of detection involving the influences of the adulterants on the color changes of the SBH honey, distinct trends can be discriminated by considering both visible and NIR regions.

**Figure 7:**
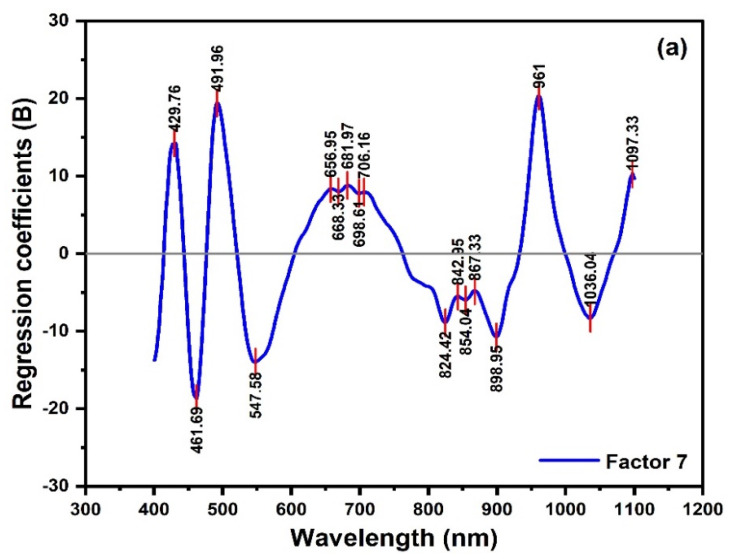

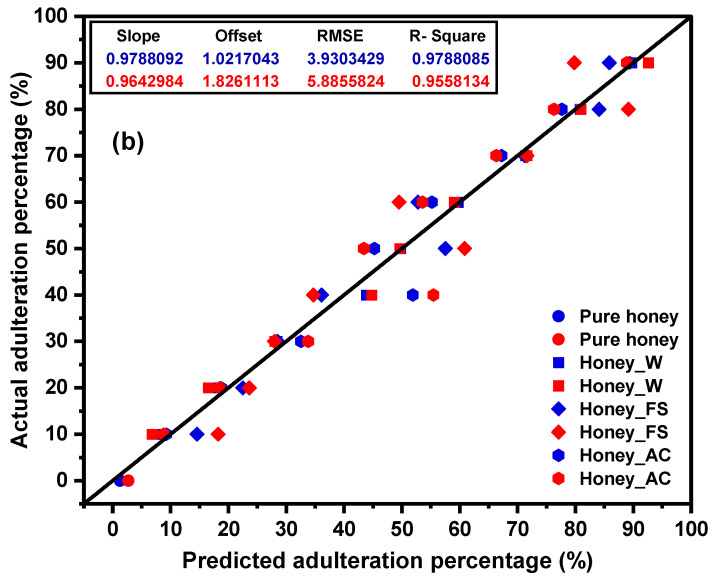
PLSR model results: (**a**) regression coefficients plot of Factor 7 and (**b**) calibration (in blue) and cross-validation (in red) at the 400–1100 nm spectral interval for SBH samples.

Table 1 summarizes the results of various pre-treatments that were applied to optimize the calibration performance of the global PLSR model utilized to quantify the level of the adulteration in the SHB samples. It is shown that $R_{C}^{2}$ and $R_{CV}^{2}$ are approximately similar for all the models examined, and therefore the model with the lowest $RMSE_{CV}$ was chosen. As displayed in Table 1, the most accurate model that produces the lowest $RMSE_{CV}$ corresponds to the moving average smoothing and the first polynomial detrending transformations using seven factors (LVs). The PLSR modeling results are presented in Figure 7. Figure 7a shows the loading plot of the regression vectors of the best PLSR model (Factor 7). The regression vectors with higher loading values displayed a high compatibility with the spectral bands presented in the PCA analysis. The positions of the spectral peaks in the regression coefficient plot of the model were marked to determine the bands that have the biggest weights in the calibration on adulterated honey. These bands characterize the diverse water structures and carbohydrates. Figure 7b demonstrated the linear regression of actual and predicted adulteration levels in the SBH samples. The value of $R_{C}^{2}$, $R_{CV}^{2}$, $RMSE_{C}$, and $RMSE_{CV}$ were 0.98, 0.96, 3.93%, and 5.88%, respectively. The obtained values of $RMSE_{C}$ and $RMSE_{CV}$ of the global PLSR model suggested its good predictive capacity and the appropriateness of the selected spectral region, 400–1100 nm.

### 2.3. Evaluation of the Structural Changes of Adulterated SBH Based on Aquaphotomics

**Figure 8:**
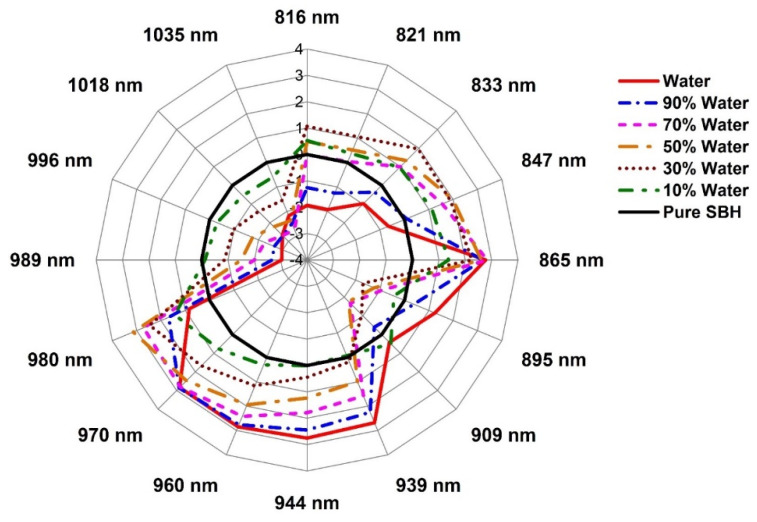
Aquagrams of pure SBH, water, and adulterated SBH with various percentages of water.

**Figure 9:**
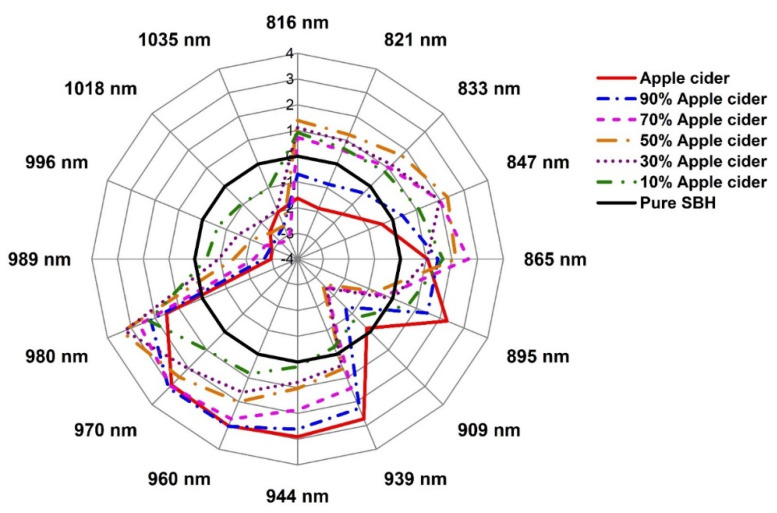
Aquagrams of pure SBH, apple cider vinegar, and adulterated SBH with various percentages of apple cider vinegar.

**Figure 10:**
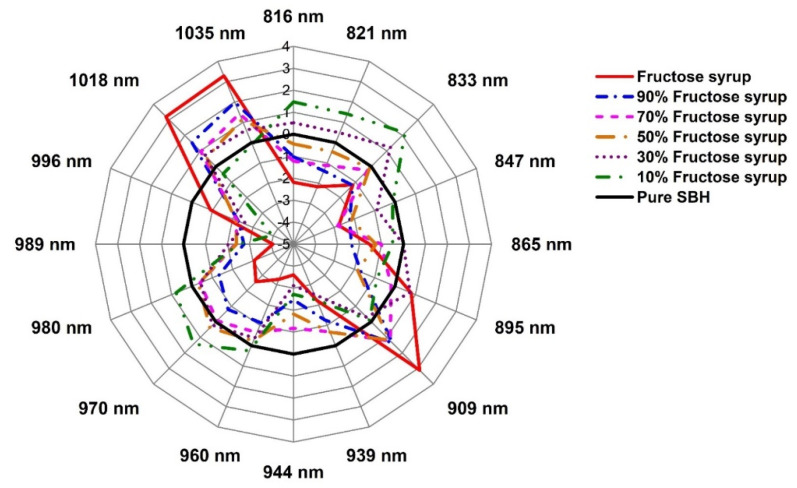
Aquagrams of pure SBH, fructose syrup, and adulterated SBH with various percentages of fructose syrup.

The aquagrams in Figure 8, Figure 9 and Figure 10 presented the structural changes in stingless bee honey after adulteration by water, apple cider, and fructose syrup, respectively. The radial axes of aquagrams are defined by 16 absorbance bands that can be considered as WAMACs. The aquagrams show WASPs of adulterated honey compared to the WASP of pure honey (the black line on Figure 8, Figure 9 and Figure 10). Clearly, the aquagrams of honey adulterated by water and apple cider (Figure 8 and Figure 9) showed similar WASPs. The first common characteristic is the decrease of the absorbance of adulterated SBH samples in the region 980–1035 nm. In this region, the higher the adulteration percentage the lower the absorbance, and this behavior can be noticed in regular steps of absorbance reduction. The lowest aquagram line in this region corresponds to pure adulterants (water and apple cider, which is a highly aqueous sample). The second common characteristic is the increase of absorbance in the region 939–970 nm, and a gradual shift towards the lower wavelengths with the increase in the percentage of adulteration. These observations are in agreement with the previous findings by Omar et al. [50] on the NIR spectral behavior of SBH adulterated with water. Particularly, it was noticed that the main NIR absorbance peak was related to water absorbance located around 950–1050 nm and that as the honey was diluted with the addition of water, the peak shifted towards lower wavelengths. Therefore, it can be concluded that the observed characteristics showed that the honey is becoming more liquid as it is more diluted by adulterants. In aquagrams, this process is represented by the restructuring of water that contains more weakly hydrogen-bonded water and free water as the percentage of adulterant is increased.

The bands 939 nm, 944 nm, 960 nm, 970 nm, and 980 nm correspond to overtones of the WAMACs located respectively at 1408.5 nm and 1416 nm (both bands can be assigned to free water molecules), 1440 nm (water dimer), 1455 nm (water hydration shell OH-(H_2_O)_4,5_ and bulk water), and 1470 nm (can be attributed to water molecules with 2 or 3 hydrogen bonds) [21]. In the region 980–1035 nm, the bands 989 nm and 996 nm can be ascribed to overtones of the WAMACs located at 1483.5 nm and 1494 nm (both bands corresponding to water molecules with 4 hydrogen bonds) [21]. The bands 1018 and 1035 nm are very close to the bands which are identified as NIR absorbance bands for water (1010 and 1030 nm) [61], while the band at 1020 nm is attributed to OH with possible H-bonding in water at 1–2 °C [62]. If it is assumed that both 1018 nm and 1035 nm bands are overtones of water bands in the first overtone of the water region, the recalculation would produce the wavelengths 1527 and 1552.5 nm, respectively. In the aquaphotomics application on honey adulteration by Bazar et al. [13], the bands 1528–1532 nm and 1564–1572 nm have high absorbance in pure honey and a decrease in a regular trend with the addition of adulterant, similar to the performance in this study. They attributed the bands above 1500 nm (in our case this would correspond to the bands above 1000 nm) to ice-like clusters of liquid water, formed by a very small amount of monomeric water species, a major part of it bound by one hydrogen bond at the periphery and two hydrogen bonds in the center. These structures represent highly organized water structures with strong H-bonds. The ice-like structures of liquid water are stable clusters with less free -OH and typically generated around macromolecules such as sugars [53]. It was shown that the addition of sugar decreases the amount of weakly H-bonded molecules, while it increases the number of H-bonded water molecules, resulting in highly organized water structures [63]. This means that these water structures include the main sugars in honey. In view of that, the bands 1018 nm and 1035 nm can be assigned to strongly hydrogen-bonded water in a crystallized form that is presented in pure honey due to the high sugar content; this water can also be thought of in terms of sugar–water interaction. The addition of water or apple cider acts on these structures as a heater, melting them and creating more weakly hydrogen-bonded water. These findings are in agreement with what was observed during the initial inspection of the raw spectra in Figure 1 and confirm that the origin of the scattering and changes in the baseline, that was so prominent in the case of adulteration by water and apple cider, are indeed due to the altered water–sugar interaction, with the increase of bulk and hydration water and changes in the crystallization of sugars in honey.

A similar decrease in absorbance at aquagrams for honey adulteration by both water and apple cider happens at the 909 nm band. This band was found to be strongly related to the sugar C-H group [51,64,65,66,67], and so it can be assigned to the sugar–water interaction. The entire 870–890 nm region can be attributed to carbohydrates [68], and the band at 895 nm may be related to some specific type in apple cider. The band at 895 nm clearly makes the difference in spectral patterns of honey adulterated by water and apple cider and is highly characteristic and presented with high absorbance in the case of apple cider. Alternatively, it may also be ascribed to the absorbance of protonated water clusters H^+^.(H_2_O)_3_ [69]. The region 816–865 nm incorporates several overlapped spectroscopic features of compounds with C-H and O-H groups, including proteins, carbohydrates, hydrocarbons, alcohols, and water [62], with water being a stronger absorber around 830–840 nm [68,70].

The aquagram of SBH adulterated with fructose syrup in Figure 10 shows a spectral pattern quite different from those of Figure 8 and Figure 9. With an increase in the percentage of fructose syrup, there is an increase in absorbance at 1018–1035 nm and 909 nm. The fructose syrup is a highly concentrated solution of sugars, and this agrees well with the assignments of these bands to the sugar–water interaction. These two features are the most prominent characteristic of honey adulterated with fructose syrup, while the absorbance shows a marked decrease at other bands. Adulteration with fructose syrup seems to obliterate the features of honey, resulting in a significant decline in absorbance at all bands, even when the adulteration is at the lowest level of 10%. Hence, it can be deduced that the adulteration of honey with fructose syrup is the most serious case compromising the honey’s structure and functionality.

From the above discussion, our findings agree with the earlier reports about the effects of the adulteration of honey. A fast and simple method for the detection of SBH adulteration was presented using aquagrams for the region 800–1100 nm, where the most common photodetectors available in the market offer the best sensitivity [51]. For the development of a simple multispectral sensor, the results suggested that the bands at 865 nm and 980 nm have the highest specificity for the detection of the adulteration of SBH by water. In the case of apple cider vinegar, the highest specificity for adulteration detection was found at the bands 865 nm, 970 nm, and 980 nm, and lastly, for adulteration by fructose syrup, the bands at 847 nm, 939 nm, 944 nm, 989 nm, and 996 nm. Therefore, using eight wavelengths (847 nm, 865 nm, 939 nm, 944 nm, 970 nm, 980 nm, 989 nm, and 996 nm), a simple and multispectral portable measurement system for detecting SBH adulteration can be developed based on the method presented in this work, that allows for the detection of adulteration and the type of adulterant.

## 3. Materials and Methods

### 3.1. Honey Samples

The pure stingless bee honey (SBH) sample was purchased from MUKAS Stingless Bee Farm, which is located in Kedah, Malaysia. All samples were harvested in the year 2018, stored in airtight jars, and refrigerated until analysis.

### 3.2. Adulterants

The three types of adulterants used in this study were distilled water, apple cider vinegar, and high fructose syrup, which are common SBH adulterants used in the market. Both apple cider and fructose syrup were purchased from Sinaran Saintifik Enterprise, Penang, Malaysia.

### 3.3. Honey Adulteration Sample Preparation

The pure SBH samples were adulterated by three different types of adulterants, which were distilled water, apple cider vinegar, and high fructose syrup. The SBH samples were adulterated with various degrees of adulteration (10%, 20%, 30%, 40%, 50%, 60%, 70%, 80% and 90%). The mixture was classified as adulterated honey, and the sample was thoroughly shaken until a homogenous mixture was formed. Prior to analysis, the honey samples were stored in glass jars, in a dark place, at room temperature. The overall number of samples was 31, comprising 1 pure SBH sample (0%), 3 adulterants (100%), and 27 adulterated honey samples (10–90%). The adulterated SBH samples consisted of 3 groups of 9 samples each, adulterated with water, apple cider vinegar, and high fructose syrup, accordingly. The adulteration level was from 10 to 90% with an interval of 10%.

### 3.4. Vis-NIR Spectroscopy

The visible and near-infrared (Vis-NIR) absorbance spectroscopy measurement of the samples was conducted using the QE65000 spectroscopic system (Ocean Optics, Inc., Dunedin, FL, USA) with a spectral range between 400 and 1100 nm. Pure and adulterated honey samples in glass holders (1 cm × 1 cm) were scanned at room temperature (~25 ℃) using the QE65000 spectrometer. The absorbance measurement of the sample is relative to an empty cuvette and was calculated using Equation (1):

(1) $$A_{\lambda }=-\log_{10}\left(\;\frac{S_{\lambda }-D_{\lambda }}{R_{\lambda }-S_{\lambda }}\;\right)\;$$

where:

$A_{\lambda }$ is the absorbance at wavelength λ

$S_{\lambda }$ is the intensity of light transmitted through the sample at wavelength λ

$D_{\lambda }$ is the dark intensity at wavelength λ

$R_{\lambda }$ is the intensity of light transmitted through the reference (empty cuvette)

### 3.5. Color Analysis

Color characteristics were determined using a Chromaticity Diagram template developed in the OriginPro 2021 Software Package (version 9.8, Origin Lab Corporation, Northampton, MA, USA). The template was designed to calculate the color parameters from the spectra according to the equations from the CIE Technical Committee [71]. The CIELAB color space was utilized [2,36,48,49], with reference to illuminant D65 and a visual angle of $10^{{}^{\circ}}$ (CIE 1964 Supplementary Standard Observer) [48,49]. The wavelength range used in the calculation was 380–780 nm [47].

### 3.6. Total Soluble Solids

The soluble solids content (SSC: ^o^Brix) of the prepared samples was measured using a PAL-3 refractometer from Atago, Co. (Tokyo, Japan) with a range of measurements from 0 to 93 ^o^Brix and a resolution of 0.1 ^o^Brix.

### 3.7. Multivariate Analysis

First, the Vis-NIR spectral data were preprocessed with some transformations to minimize unwanted effects, that include baseline variation, light scattering, and path length variances [72]. A moving average smoothing with a segment size of 25 was performed to eliminate random variations of the data set and correct the scatter effect. Baseline correction was used to adjust the spectral offset. The first-order detrending transformation was used to remove the baseline of the signals in the spectral data [73] and detach the particular absorption features [74]. The first derivative order of Savitzky–Golay (SG) with 25 smoothing points was utilized to reduce the baseline variation and augment the spectral features [75].

A principal component analysis (PCA) was applied to study the clustering of the samples. The three-dimensional (3D) score plot and the corresponding loadings of the principal components (PCs) were presented for the pre-processed spectra. In addition, the partial least squares regression (PLSR) model was developed to quantify the adulteration level in honey samples. The regression model was validated by the full cross-validation method. The precision and accuracy of the PLSR model was evaluated by the determination coefficient ($R^{2}$) and the root mean square error (RMSE) of calibration and cross-validation ($R_{C}^{2}$, $R_{CV}^{2}$, $RMSE_{C}$, $RMSE_{CV}$). A good model should have a high $R^{2}$ and a low RMSE. The PLSR models with 1–10 factors (latent variables ‘LVs’) were examined, and the optimal number of LVs was selected based on the lowest value of the $RMSE_{CV}$. Both PCA and PLSR were performed using the Unscrambler Software (version 10.4, CAMO Software AS, Oslo, Norway).

### 3.8. Aquagrams

An aquaphotomics analysis was applied to examine the influence of each adulterant depending on the concentration. The raw spectra were pre-processed using Savitzky–Golay’s second-order polynomial smoothing with 21 points to remove noise, linear baseline correction to correct the baseline slope, and normal variate transformation (SNV) to remove the baseline offset. These pre-processing methods were applied on datasets split according to the adulterant type, because of the specific effects of each adulterant on the baseline. First, the loadings of PCA and regression coefficients of PLSR were inspected for consistently appearing and highly influential variables, that can be considered as Water Matrix Coordinates (WAMACs). Based on this evaluation, the wavelength region from 800 to 1100 nm was opted for aquaphotomics analysis, where 16 important absorbance bands were selected to be presented on the aquagrams. Afterwards, the absorbance values were normalized following the procedure for calculation of classic aquagrams [23], and the spectrum of pure honey was subtracted to allow for the comparison with the adulterated honey samples. The water absorbance spectral patterns (WASPs) of honey with each additive (water, apple cider, and syrup) were presented on aquagram plots featuring normalized absorbance and displayed on radar axes defined by selected WAMACs. Pre-processing was performed using the commercially available software Pirouette 4.0 (Infometrix Inc., Bothell, WA, USA), and the aquagrams were prepared in Microsoft Office Excel 2016 (Microsoft Co., Redmond, WA, USA) and depicted using the OriginPro 2021 Software Package (version 9.8, Origin Lab Corporation, Northampton, MA, USA).

## 4. Conclusions

The characteristics of pure and adulterated SBH were studied using visible (Vis) and near-infrared (NIR) spectroscopy. The SBH was adulterated with various adulterants, including distilled water, apple cider vinegar, and high fructose syrup, with a percentage ranging from 10% to 90%. The visible peak of the SBH was altered, but the shift in the visible peak was apparent only for the level of adulterants beyond 50%. On the other hand, the shift in NIR is only significant for SBH adulterated with water and apple cider, contributed by the change in the water structure of the samples. The classification of the adulterants and both pure and adulterated SBHs was attained using a principal component analysis (PCA). A partial least squares regression (PLSR) global model was developed to quantify the adulteration level in the honey samples. An optimum PLSR prediction model with $R_{CV}^{2}=0.96$ and $RMSE_{CV}=5.88\;\%$ was acquired. In conclusion, Vis-NIR absorbance spectroscopy at the region 400–1100 nm has proven a promising method in identifying the adulterated SBH. The alteration in the spectral attribute, however, is highly dependent on the nature of the liquid added to the pure SBH. Furthermore, the results showed that the region at 400–1100 nm that is related to the color and water properties of the samples is effective to discriminate and quantify the adulterated SBHs.

For the first time, the adulteration of SBH has been investigated using the combination of the spectroscopy technique and the aquaphotomics approach at the short-wavelength NIR region (800–1100 nm). The adulteration level in SBH including 10% was identified, irrespectively of the adulterant type. It was found that each adulterant resulted in a change in the spectral pattern of honey in a unique way that can be explained by the interaction of the adulterant with the chemical structure of honey. This method can be applied for developing highly sensitive and portable measurement systems for detecting adulterated SBH.

## Figures and Tables

**Table 1:** Calibration and cross-validation results of the PLSR model performed on pre-processed spectral data at the range 400–1100 nm.

Pre-Processing Methods	LVs	$RMSE_{C}\;(\%)$	$RMSE_{CV}\;(\%)$	$R_{C}^{2}$	$R_{CV}^{2}$
Raw absorbance	7	3.65	6.43	0.98	0.95
Smoothing	7	4.20	6.49	0.97	0.95
Detrend 1st polynomial	7	2.34	6.33	0.99	0.95
1st derivative SG	6	3.85	6.29	0.98	0.95
**Smoothing, Detrend 1st Polynomial**	**7**	**3.93**	**5.88**	**0.98**	**0.96**
Smoothing, detrend 1st polynomial, 1st derivative SG	6	4.08	6.58	0.98	0.94

## Data Availability

Not avalilable.

## Supplementary Materials

The following supporting information can be downloaded at: https://www.mdpi.com/article/10.3390/molecules27072324/s1, Figure S1. CIE x, y chromaticity diagram showing the variation of color of the samples (*n* = 31). Figure S2. Zoom-in view of CIE x, y chromaticity diagram of the samples (*n* = 31). Figure S3. 2D scores plot of PC1 vs. PC2 of the samples (*n* = 31). Figure S4. 2D scores plot of PC1 vs. PC3 of the samples (*n* = 31). Figure S5. 2D scores plot of PC2 vs. PC3 of the samples (*n* = 31).

## Sample Availability

Samples of the compounds are not available from the authors.
