# Supplementary material for: Identification of Stingless Bee Honey Adulteration Using Visible-Near Infrared Spectroscopy Combined with Aquaphotomics
Source: Molecules. 2022 Apr 3;27(7):2324. doi: 10.3390/molecules27072324 (PMC9000493; doi:10.3390/molecules27072324)
Supplement: Supplementary file 1 [file molecules-27-02324-s001.zip › molecules-1629506-supplementary.pdf]

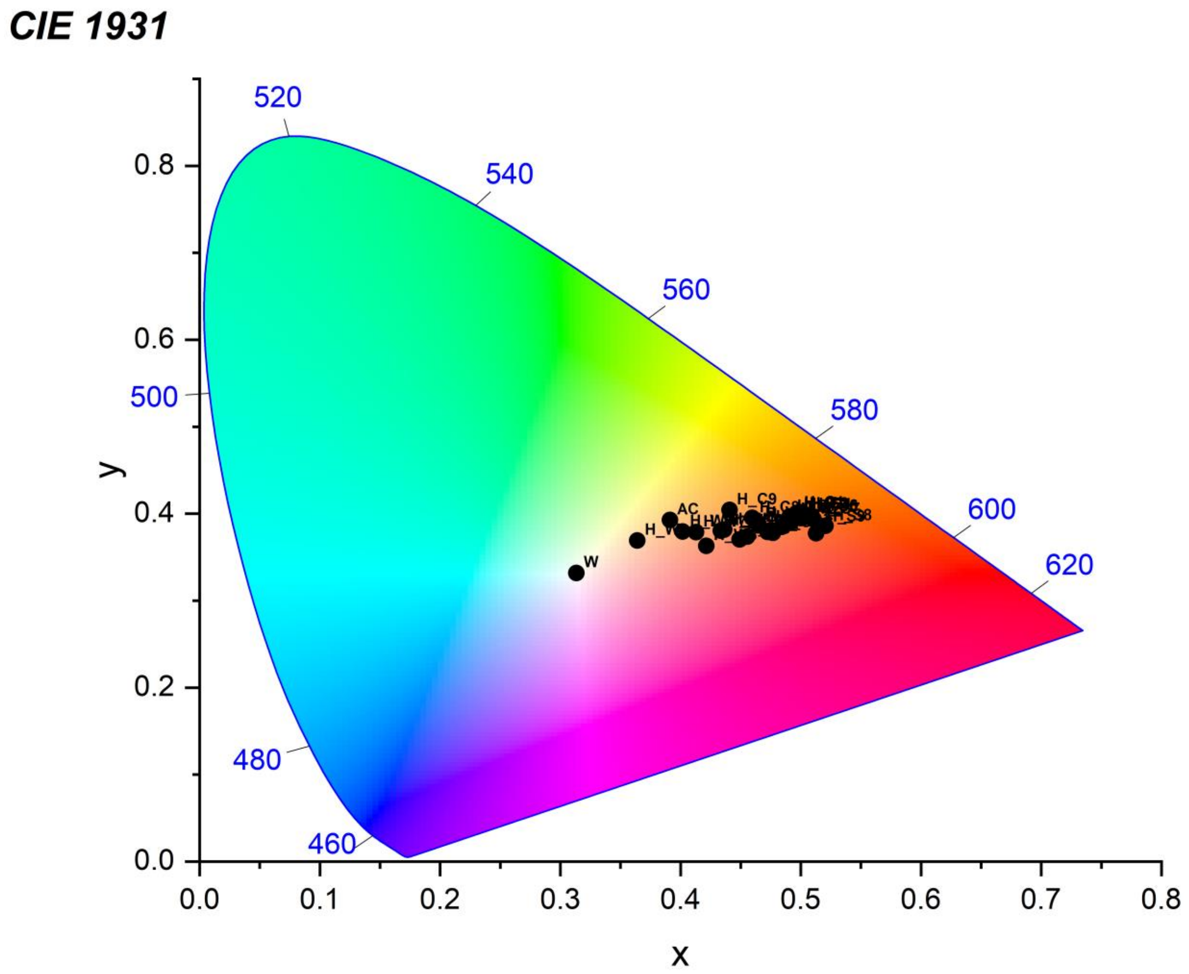

Figure S1. CIE x, y chromaticity diagram showing the variation of colour of the samples (n = 31).

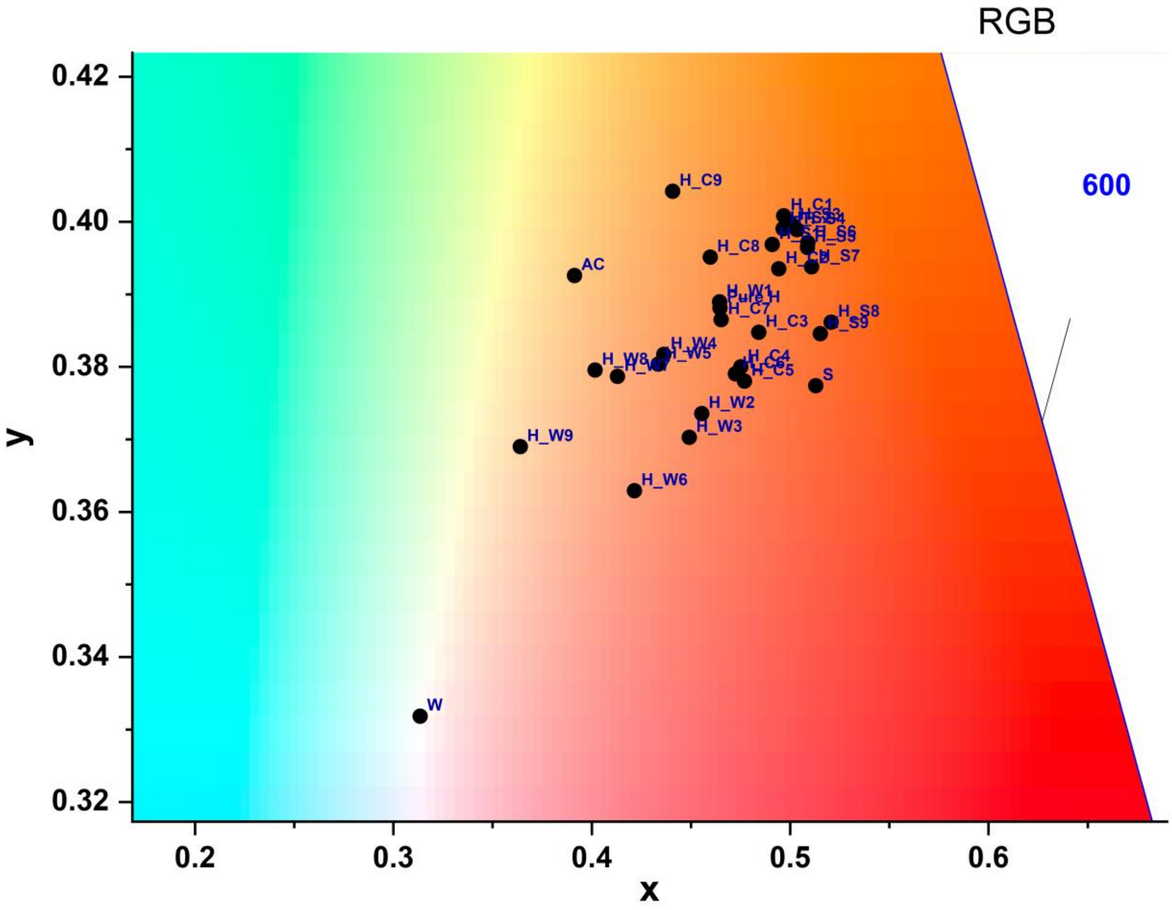

Figure S2. Zoom-in view of CIE x, y chromaticity diagram of the samples (n = 31).

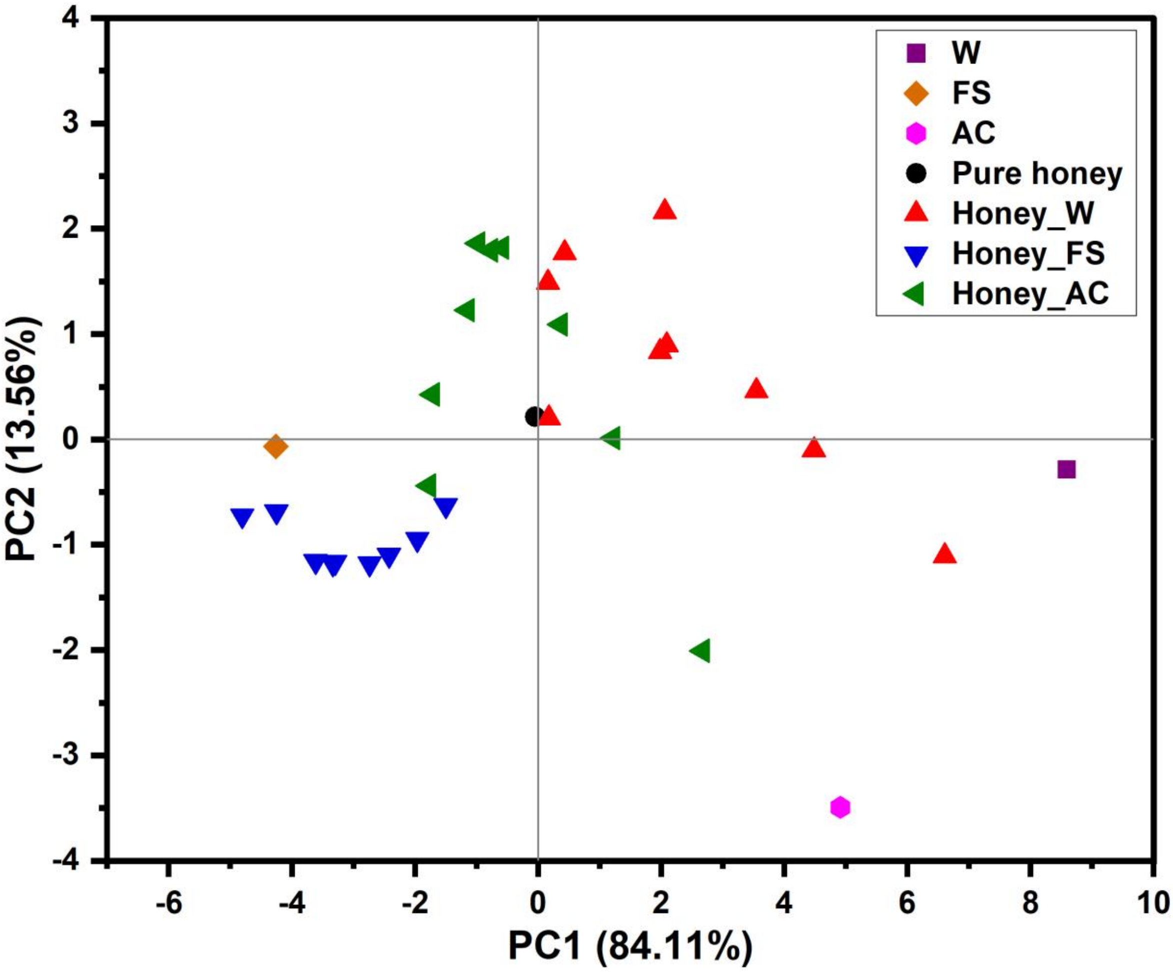

Figure S3. 2D scores plot of PC1 vs. PC2 of the samples (n = 31).

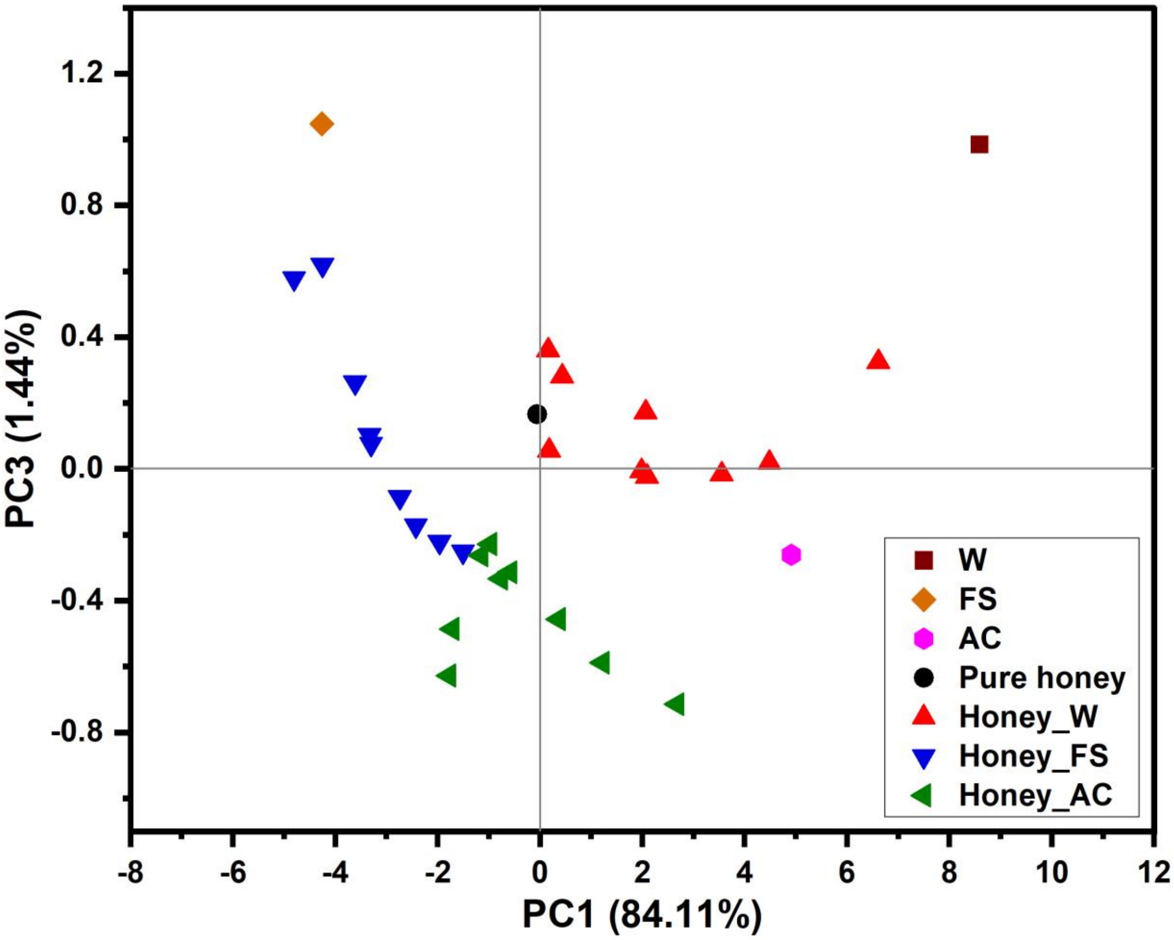

Figure S4. 2D scores plot of PC1 vs. PC3 of the samples (n = 31).

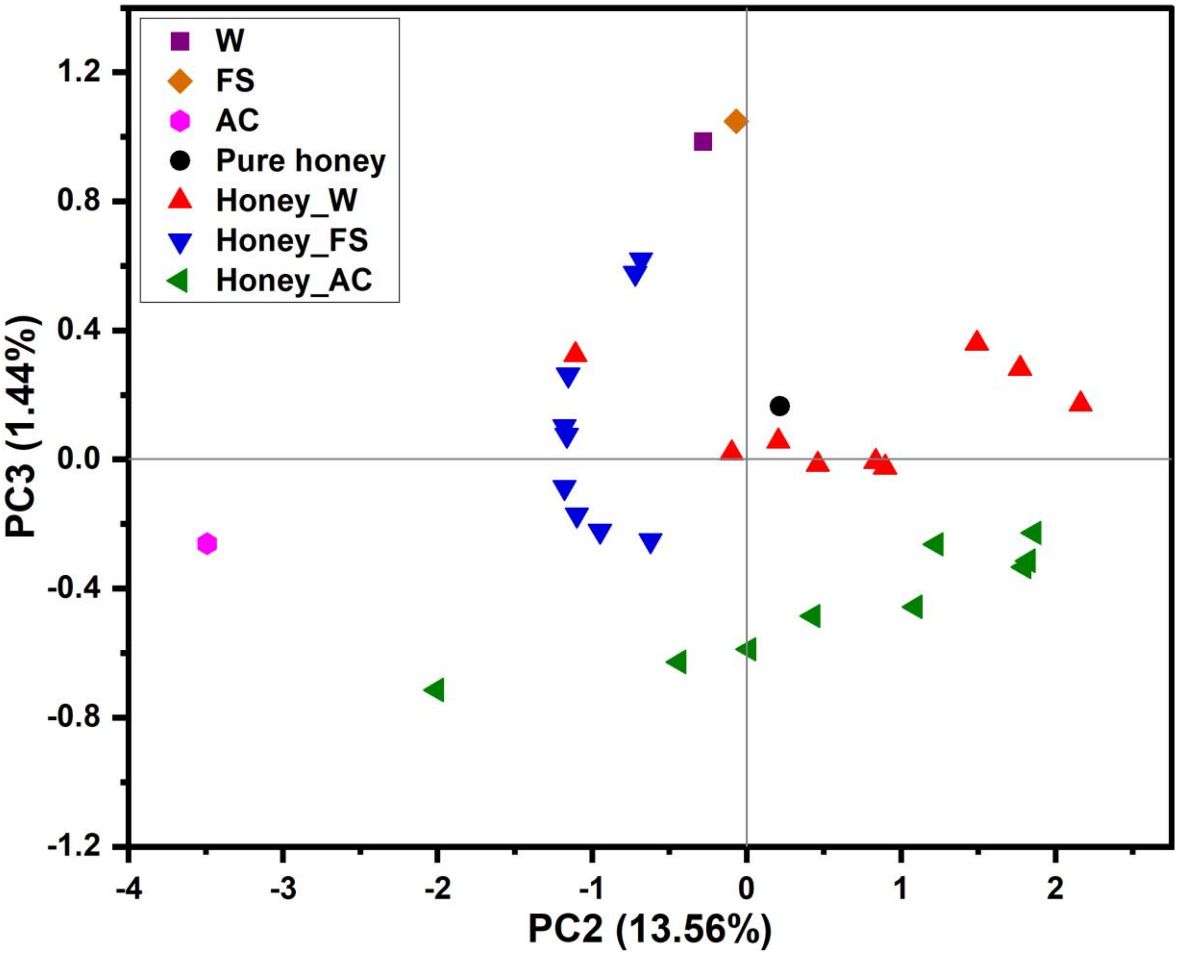

Figure S5. 2D scores plot of PC2 vs. PC3 of the samples (n = 31).
